# Managing Temporomandibular Joint Ankylosis Concurrent With Extrahepatic Portal Vein Obstruction: A Report of a Rare Case and Literature Review Investigating the Hypercoagulability Link

**DOI:** 10.7759/cureus.54478

**Published:** 2024-02-19

**Authors:** Subhasish Burman, Asish K Das, Aquila A Anwar, Abhijit Maji, Abhishek Khatua

**Affiliations:** 1 Oral and Maxillofacial Surgery, Dr. R. Ahmed Dental College and Hospital, Kolkata, IND

**Keywords:** temporomandibular ankylosis, interpositional arthroplasty, extrahepatic portal vein obstruction (ehpvo), extrahepatic portal vein obstruction, temporomandibular joint ankylosis

## Abstract

This report describes the understudied co-occurrence of temporomandibular joint ankylosis (TMJA) and extrahepatic portal vein obstruction (EHPVO), exploring a shared pathway involving hypercoagulability. TMJA is an acquired pathology where joint surfaces fuse, causing restricted mouth opening and facial asymmetry. Globally, TMJA is prevalent among 1.5 to 5 patients/million, with a higher incidence in developing countries. While trauma and infections often cause TMJA, the pathogenesis remains unclear in many cases. Recent literature notes a link between TMJA and EHPVO, a noncirrhotic vascular disorder causing portal hypertension and upper gastrointestinal bleeding in children. Prothrombotic disorders such as protein C and S deficiency may contribute to EHPVO, mirroring TMJA's association with hypercoagulability. This report focuses on an 11-year-old female diagnosed with TMJA, accompanied by a history of ear infection and concurrent EHPVO. We further presented clinical observations, surgical interventions, and outcomes alongside a literature review to understand the probable connection between EHPVO and TMJA.

## Introduction

Temporomandibular joint ankylosis (TMJA) involves pathological changes affecting the structure and function of joint surfaces. Although there is a lack of comprehensive studies on the prevalence of TMJA at various geographic levels, Gupta et al. found a prevalence of 0.46 per 1000 among three to 15-year-olds in rural and urban areas of Lucknow, India, in 2012 [1]. TMJA can manifest at any age, resulting in restricted mouth opening and facial asymmetry [2]. The prognosis is seen to be negatively correlated with the number of years with TMJA. In 1982, Rowe identified various etiological factors, including trauma being the most prevalent cause (31-98%), followed by local or systemic infections (10-49%) and systemic diseases (10%) [3].

The pathogenesis of ankylosis has been explored through diverse hypotheses over the years. The Interfragmentary Strain Theory, also known as Perren's Strain Theory, posits that minimal strain or limited jaw movement post-trauma induces endochondral ossification, particularly in the lateral aspects of the joint [4]. Ferretti et al. proposed the Hemarthrosis Theory, suggesting a role for hemarthrosis in ankylosis formation [5]. Meng et al. linked forces akin to distraction osteogenesis, exerted by the lateral pterygoid on sagittal condyle fracture healing, to ankylosis. Genetic predisposition reconsidered by Hall and the postulation by Bhatt et al. regarding the potential contribution of hypercoagulability of blood in TMJA add complexity to the current understanding of this condition [5]. While it is widely acknowledged that ankylosis results from the organization of a hematoma following condylar trauma, evidence indicates that only a small percentage (0.2% to 4%) of condylar fractures ultimately advance to ankylosis [5,6]. Recently, the hypercoagulability theory postulated by Bhatt et al. was tested through a case-control evaluation by Roychoudhury and team. They observed that hypercoagulability, in conjunction with other factors such as age below 10 years, lack of timely treatment, and female gender, acted as a potentiating factor for post-traumatic TMJA. Bhatt et al. observed the coexistence of extrahepatic portal vein obstruction (EHPVO) in the cases they described [4,5].

EHPVO is a noncirrhotic vascular disorder affecting the portal vein outside the liver. In older children, conditions such as abdominal infections (e.g., peritonitis), primary sclerosing cholangitis, chronic pancreatitis, myeloproliferative disorders, and hypercoagulable disorders like factor 5, protein C, and protein S deficiency can lead to EHPVO [7]. Hepatic complications encompass portal hypertension, presenting as splenomegaly, ascites, abdominal pain, and severe upper gastrointestinal bleeding, and may progress to jaundice, coagulopathy, and fatal hepatic encephalopathy [8]. Oral and cutaneous manifestations include spider angiomas, gingival bleeding, mucosal telangiectasia, and palmar erythema [9]. Recent literature indicates an association between EHPVO, secondary to hypercoagulable disorders, and the incidence of TMJA [5]. This article intends to present one such case report alongside an existing literature review. Further, we have provided insight into the course and treatment outcomes adopted to treat TMJA associated with EHPVO.

## Case presentation

**Figure 1:**
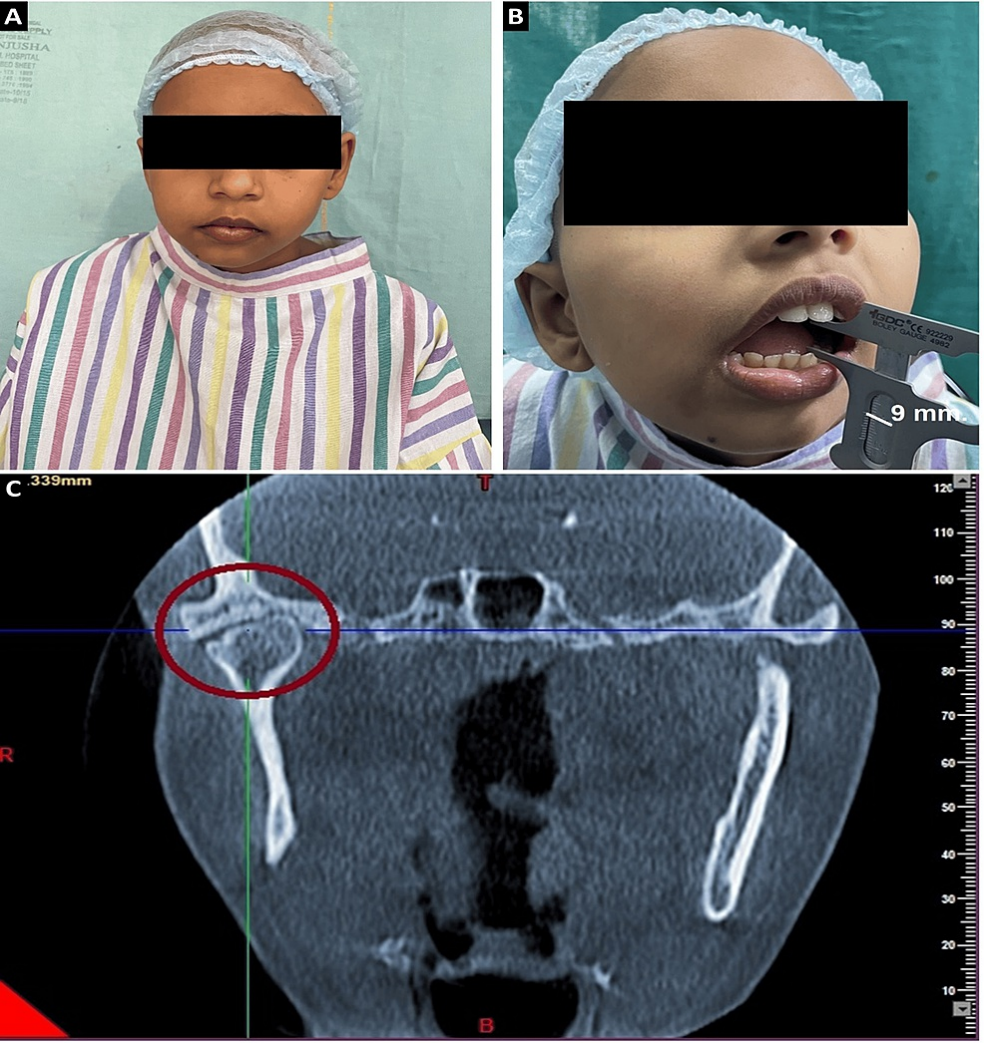
Pre-operative clinical and radiographical evaluation of the patient (A) Patient's frontal profile (B) Mouth opening limited to 9 mm with deviation of chin towards the right side (vernier caliper readings indicated by a white marker) (C) Coronal section obtained from cone beam computed tomography (CBCT), highlighting Sawhney’s Type I right temporomandibular joint ankylosis (TMJA). The ankylosed joint is delineated by a red circle for identification and reference

An 11-year-old female patient was referred to our department of Oral and Maxillofacial Surgery, Dr. R. Ahmed Dental College and Hospital, Kolkata, with a history of gradual reduction of mouth opening for the last one and half years following an infection in the right ear. The patient's medical history indicated a confirmed case of EHPVO, undergoing symptomatic treatment for recurrent hematemesis and melena, with management involving a daily regimen of 10 mg non-selective beta-blocker propranolol. Examination revealed restricted mouth opening (9 mm). There was mild facial asymmetry and deviation of the chin towards the right side (Figures 1A-1B). General examination revealed mild tachycardia. Per-abdominal examination revealed tenderness and mild splenomegaly. On pre-auricular palpation, restricted movements of the joint on the right side were noted. Clinical and radiological evaluation confirmed Sawhney’s Type I unilateral right-sided ankylosis (Figure 1C). Hemoglobin (Hb) level was 9 gm/dl, and total leucocyte count was 3200/cumm. Platelet count was 1 lakh/cumm. On the coagulation profile, protein C levels were mildly decreased to 42 IU/dl (the normal range in healthy adults is 65 to 135 IU/dl). Renal function and liver function tests, pancreatic enzymes, and blood glucose levels were within normal limits.

A discussion was done with the Department of General Medicine, Nil Ratan Sircar Medical College and Hospital, Kolkata, where the patient was receiving treatment for EHPVO. The age of onset of ankylosis was around nine years, i.e., after a prepubertal growth spurt. So there was mild facial asymmetry for two years. In adherence to the ankylosis treatment protocol, the decision was made to release the ankylotic mass and incorporate an interpositional material. This would restore normal mouth opening, balance the ramus-condyle length discrepancy, and improve the patient’s malnutrition, which will help in subsequent endoscopic surgery of variceal ligation. Following the release of ankylotic mass, some correction of resultant asymmetry will also be possible when the deficient side of the mandible undergoes growth and development in the residual pubertal growth phase as per Melvin Moss’s Functional Matrix theory. Meanwhile, the patient was kept on a non-selective beta-blocker and anticoagulants for symptomatic relief.

Therapeutic intervention

**Figure 2:**
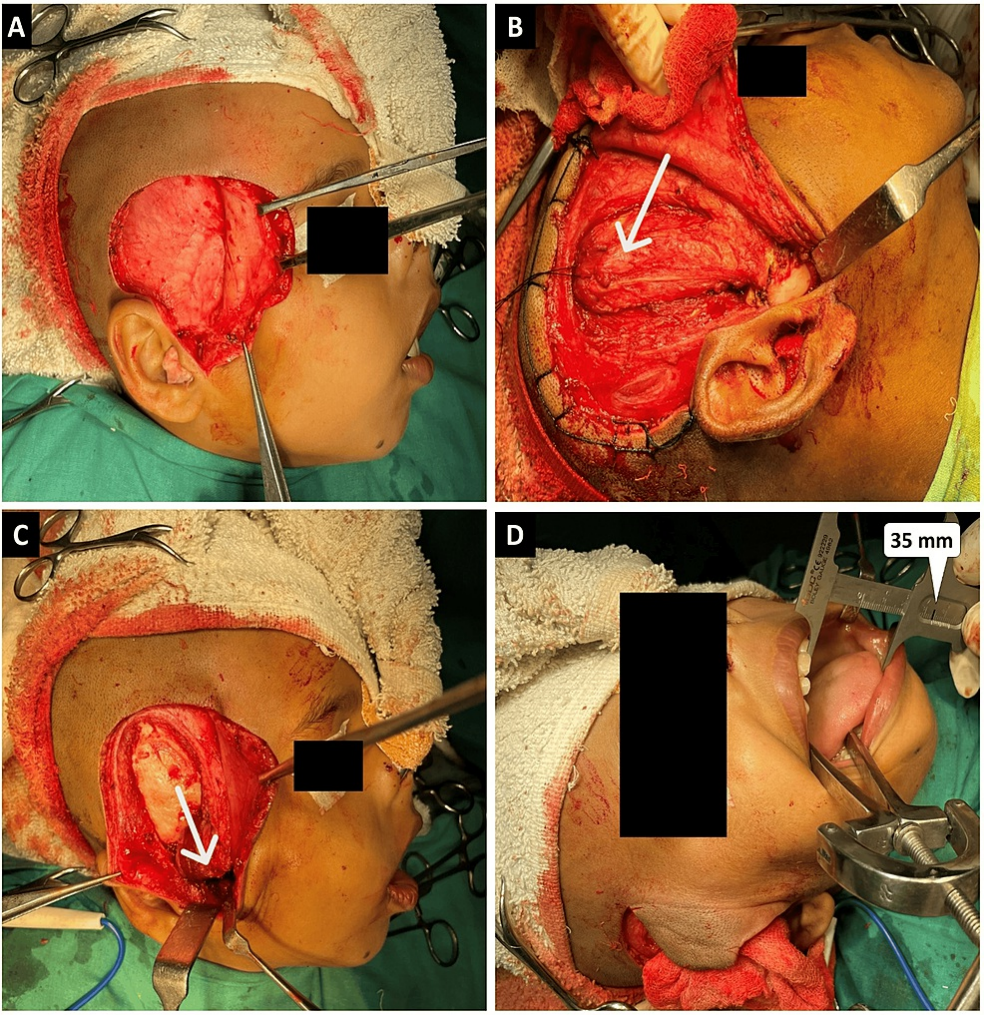
Surgical procedure (A) Al Kayat and Bramley's modification of the preauricular incision, revealing the exposed temporoparietal fascia following dissection (B) White arrow shows the harvesting of the temporalis myofascial pericranial flap following the dissection of the ankylosed mass (C) Interpositioning of the flap into the created gap (marked with a white arrow) and subsequent suturing (D) On-table mouth opening of 35 mm (vernier caliper readings indicated by a white marker)

The thyromental distance was 6 cm. Also, TMJA caused a shift in the position of the larynx. Keeping the difficulty of intubation and the chances of bleeding in mind, fiberoptic intubation was initiated. The tracheostomy was kept on standby. Following confirmation of intubation (via end-tidal carbon dioxide (ETCO_2_) and auscultation of both sides of the chest), induction of anesthesia was initiated. The incision site on the right was marked with a surgical marker. The surgical site was prepared and infiltrated with a tumescent solution. Al Kayat and Bramley's modification of the preauricular incision was made on the right side (Figure 2A). This incision extended from the inferior attachment of the tragus, traversing the helix, and reaching up to the superior attachment of the lobule of the ear. In the temporal region, a question mark-shaped incision, equivalent in length to the pinna, was made into the hairline anterosuperior to the pinna, thus avoiding branches of superior temporal vessels. A deep incision was made through the superficial fascia and subsequent blunt dissection was performed to establish an avascular plane extending from the perichondrium of the tragus. The dissection proceeded anteroinferiorly, creating separation within the fascia enveloping the posterior aspect of the parotid glenoid lobe and the perichondrium of the cartilaginous section of the external acoustic meatus. Within the temporal region, similar blunt dissection was done below the superficial fascia to the level of temporal fascia, facilitating the protection of facial nerves and vessels with the reflection of the flap (Figure 2A). A 45-degree incision was made from the root of the zygoma, extending to 2 cm above the arch where the temporal fascia divides. With subperiosteal dissection over the zygomatic arch, the periosteum and superficial layer of temporal fascia were reflected together. The ankylosed side on the right was exposed. Malleable copper retractors were placed to prevent damage to the branches of the maxillary artery. Utilizing a fissure bur and osteotome, the ankylosed segment was excised in a piecemeal fashion. Subsequently, an ipsilateral coronoidectomy was done. An on-table mouth opening of 35 mm was achieved (Figure 2D). A pedicled, inferiorly based temporalis myofascial flap was harvested, repositioned, and sutured within the 1.5 cm gap resulting from the excision of the ankylosed mass, using absorbable 3-0 vicryl suture (Figures 2B-2C). Mini Vac size 8 suction drain (Romsons Scientific and Surgical Pvt. Ltd., Agra, India) was placed. After achieving hemostasis, the surgical site was closed in layers using 2-0 vicryl, 3-0 vicryl, and 3-0 prolene sutures. The patient was extubated uneventfully.

**Figure 3:**
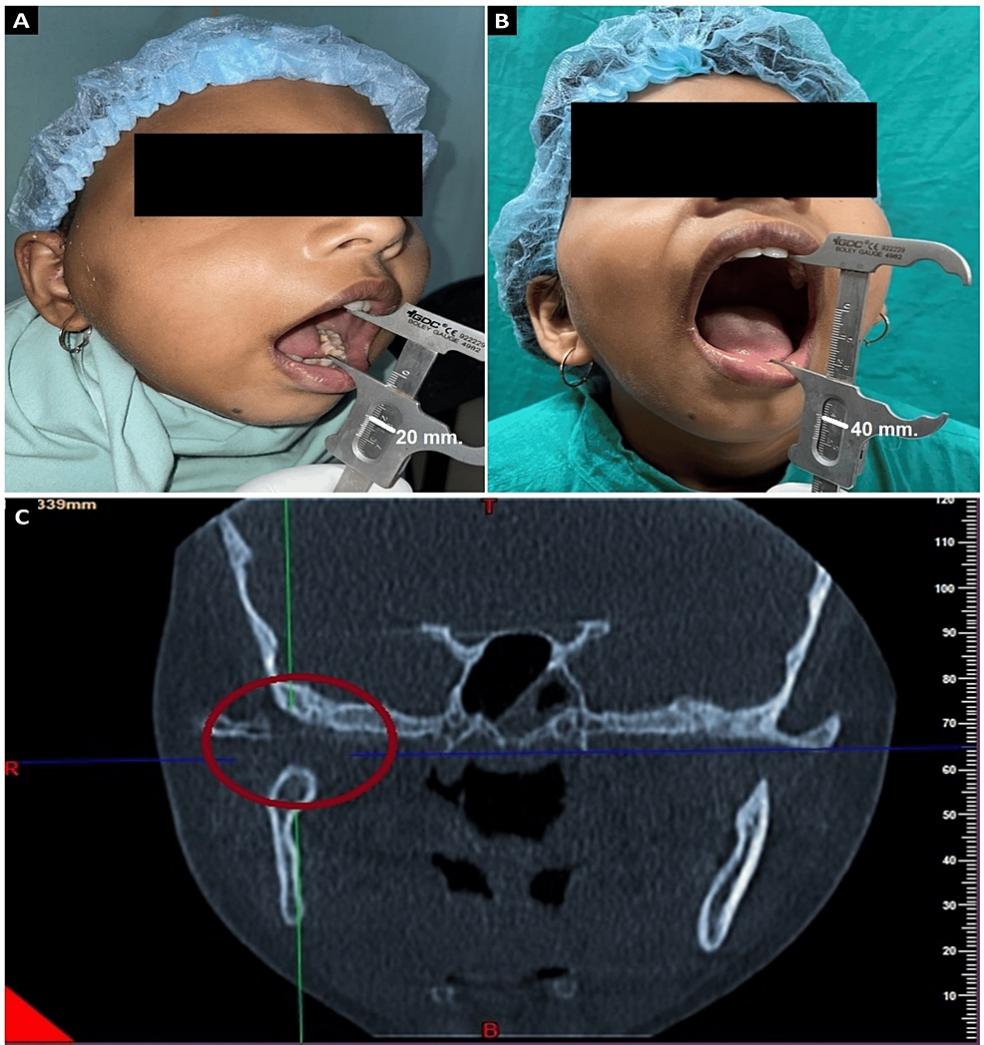
Post-operative evaluation of the patient at different follow-up periods (A) Observation of a 20 mm mouth opening at the one-week follow-up (vernier caliper reading marked with white marker) (B) Observation of a 40 mm mouth opening at the one-year follow-up (C) Coronal section obtained from cone beam computed tomography (CBCT), highlighting no heterotopic bone formation or re-ankylosis

Follow-up appointments were scheduled for the patient at week 1, month 1, month 3, month 6, and one year post the surgical intervention. Post-operative mouth opening was 20 mm in the first week (Figure 3A). With persistent physiotherapy, mouth opening was 22 mm in the third month. A 40 mm mouth opening was achieved after one year as can be seen in Figure 3B. Post-operative cone beam computed tomography (CBCT) was done at the one-year follow-up. It showed the gap created during the release of ankylosis was maintained and there was no heterotopic bone formation or re-ankylosis (Figure 3C).

## Discussion

Understanding of the temporomandibular joint and its ankylosis has evolved over the period. Recent literature, genetic, and molecular studies further elaborate on the multifactorial origin of this disease. There is a likely correlation between hypercoagulable disorders and TMJA. We conducted a thorough literature search to explore the existing evidence indicating a probable link between TMJA and EHPVO. Medline, PubMed Central (through PubMed), and Scopus databases were searched by utilizing MeSH subject headings and indexed keywords. We included two digital databases adhering to the minimum requirement for literature review charted out in Assessment of Multiple Systematic Reviews (AMSTAR) guidelines [10]. In conducting this review, rigorous inclusion criteria were taken to encompass various study types, including randomized controlled trials, cohort studies, case reports/series, and reviews published in the English language. No date restriction was applied. We excluded non-peer-reviewed evidence/studies. The combination of the following search themes was used: ("Portal venous obstruction" OR "extrahepatic portal vein obstruction" OR ehpvo OR "portal vein obstruction" OR hypercoagulable OR obstruction) AND ("temporomandibular ankylosis" OR "ankylosis of the temporomandibular joint" OR "tmj ankylosis" OR “temporomandibular joint ankyloses” OR "tmj ankylosis" OR tmja).

Though evidence was scarce, a total of 145 articles were identified. Following the elimination of duplicates, screening of titles and abstracts was carried out, followed by a thorough assessment of full-text articles. Finally, five articles were retained based on their relevance to this case report. Table 1 compiles the existing literature that describes cases where either EHPVO or hypercoagulability as a predisposing factor for EHPVO was present, alongside the co-occurrence of TMJA. While Sahoo et al. first reported the co-occurrence of EHPVO and TMJA in a case report in 2006, the hypothesis suggesting this association was proposed later through a sequential assessment of a case series conducted by Bhatt et al. in 2013 [5]. Their proposed explanation suggests that hypercoagulability and decreased fibrinolysis in EHPVO patients lead to hematoma formation, subsequently triggering neo-angiogenesis and further contributing to TMJA. In a case-control study conducted by Roychoudhury et al. in 2015, it was found that among patients with TMJA selected as cases, there was a significantly higher prevalence of hypercoagulability (p-value 0.01) compared to controls [9]. However, it is important to note that the study's sample size was limited, necessitating the need for future investigations in terms of adjusting potential confounding factors and establishing a more conclusive association.

**Table 1:** Evidence table summarizing existing literature on temporomandibular joint ankylosis and extrahepatic portal vein obstruction TMJA: temporomandibular joint ankylosis, EHPVO: extrahepatic portal vein obstruction, NA: not available

Study Details	Type of Study	No. of Cases	Age (Years)	Description	Therapeutic Intervention
Current case	Case Report	1 (female)	11	Unilateral right-sided TMJA with a prior history of ear infection and a confirmed diagnosis of EHPVO; Blood work showed a mild decrease in protein C level to 42 IU/dl	Interpositional arthroplasty using temporalis muscle flap
Sahoo et al. 2006 [11]	Case Report	1 (male)	8	TMJA with a known case of EHPVO with portal hypertension, esophageal varices, and splenomegaly for the last seven years; Hypercoagulability was not assessed	Interpositional arthroplasty using temporalis muscle flap
Bhatt et al. 2013 [5]	Case Series	4 (2 male and 2 female)	7-30	TMJA etiology was trauma; Two cases were protein C deficient, and the other two randomly selected TMJA patients were resistant to activated protein C	NA
Roychoudhury et al. 2015 [9]	Case-control Study	Case: 41 (TMJA); Control: 36 (condylar fracture follow-up patients with no ankylosis)	NA	Assessed the association between hypercoagulability and TMJA occurrence; Significant hypercoagulability was found among the cases (21.9% in cases vs. 2.8% in controls with a p-value of significance 0.01)	NA
Akshat et al. 2017 [12]	Letter to Editor	1 (male)	9	TMJA with obstructive sleep apnoea and a case with esophageal varices with EHPVO, portal hypertension, and splenomegaly for the last six to seven years; Hypersplenism was noted; Blood work showed a mild decrease in protein C	Proximal lieno‑renal shunt surgery
Kurdia et al. 2021 [13]	Case Report	1 (female)	14	Hypersplenism and blood workup showed mildly decreased protein C and S; A case of recurrent esophageal variceal bleed due to EHPVO with associated TMJA	Proximal lieno‑renal shunt surgery

**Figure 4:**
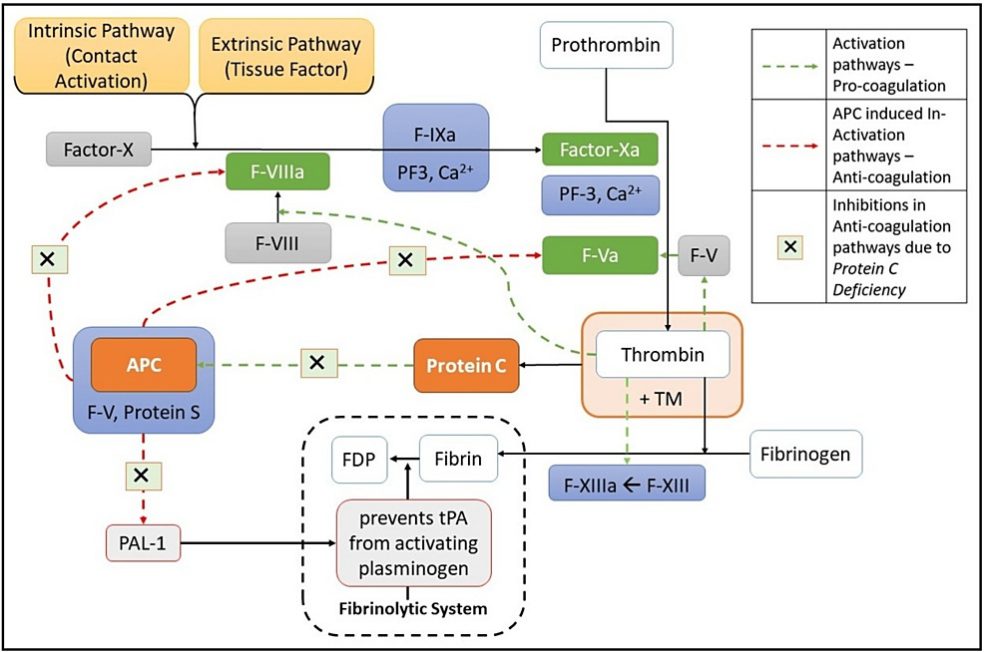
Conceptual framework depicting inhibition and inactivation of factors in anti-coagulation pathways due to protein C deficiency leading to hypercoagulability TM: thrombomodulin, PF: platelet factor, FDP: fibrin degradation product, APC: activated protein C, tPA: tissue plasminogen activator, PAL: plasminogen activator inhibitor Image credit: Aquila Aini Anwar

Mild to moderately deficient protein C was a consistent finding among most reports found earlier, as depicted in Table 1 [5,9,11-13]. Protein C plays a vital role in the anticoagulation processes in the human body. Injury triggers coagulation and consequent thrombin formation. Thrombin, a potent procoagulant, once binds with thrombomodulin (TM) abundant in vascular endothelium, loses its properties, and this thrombin-TM complex rapidly activates protein C [14]. The activated form of protein C (APC), along with protein S and other cofactors, inactivates procoagulant factors Va and VIIIa, thereby limiting their ability to further amplify clot formation. When there is a deficiency in protein C and S, procoagulant factors become more prominent [15]. Additionally, activated protein C (APC) plays a role in inhibiting plasminogen activator inhibitor-1 (PAI-1). In a state of protein C deficiency, PAI-1 remains uninhibited in the fibrinolytic system. PAI-1 binds to tissue plasminogen activator (tPA), forming a stable complex that hinders tPA from activating plasminogen. This inhibition of fibrinolysis effectively maintains stable clots [16]. Figure 4 describes a conceptual flowchart of how deficient protein C contributes to hypercoagulability.

Additionally, protein C possesses anti-inflammatory properties, and its deficiency contributes to prolonged inflammatory symptoms, resulting in reduced joint movement as patients seek to avoid pain and discomfort [5,16]. This indirectly amplifies the risk of ankylosis due to immobility. Further, in the case report by Kurdia et al. [13], protein S was also found deficient in the blood workup of the patient, contributing to the coagulation abnormalities in this context. Protein C can be reduced in either genetic or acquired conditions. The level of protein C is found to be as low as 35% in newborns and healthy infants [17]. Thereby, trauma-induced ankylosis (e.g., trauma during forceps delivery) is generally found to be highly prevalent in this age group.

## Conclusions

Emerging evidence indicates that the characteristic hypercoagulability and diminished fibrinolysis associated with EHPVO may contribute to hematoma formation and subsequent TMJA. However, the causative link between TMJA and EHPVO/hypercoagulability remains a subject of debate due to limited research and small sample sizes in existing studies. Comprehensive investigations involving larger cohorts and controlled designs are necessary to establish a more definitive association and explore optimal management strategies for patients with this unique intersection of conditions.

This case report underscores the significance of considering non-hepatic consequences of EHPVO and investigating underlying prothrombotic states in patients presenting with TMJA. The timings and sequence of multidisciplinary surgical modalities provided here can also further help in staging surgical protocols for such clinical conditions in the future.
